# On the Origins of Diffusion MRI Signal Changes in Stroke

**DOI:** 10.3389/fneur.2020.00549

**Published:** 2020-06-30

**Authors:** Stephen J. Blackband, Jeremy J. Flint, Brian Hansen, Timothy M. Shepherd, Choong H. Lee, Wolfgang J. Streit, John R. Forder

**Affiliations:** ^1^Department of Neuroscience, University of Florida, Gainesville, FL, United States; ^2^McKnight Brain Institute, University of Florida, Gainesville, FL, United States; ^3^Center for Structural Biology, University of Florida, Gainesville, FL, United States; ^4^National High Magnetic Field Laboratory, Tallahassee, FL, United States; ^5^Center of Functionally Integrative Neuroscience, Aarhus University, Aarhus, Denmark; ^6^Department of Radiology, New York University School of Medicine, New York, NY, United States; ^7^Department of Biomedical Engineering, University of Florida, Gainesville, FL, United States; ^8^Department of Radiology, University of Florida, Gainesville, FL, United States

**Keywords:** stroke, diffusion, glial cells, magnetic resonance (MR) imaging, magnetic resonance (MR) microscopy

## Abstract

Magnetic resonance imaging (MRI) is a leading diagnostic technique especially for neurological studies. However, the physical origin of the hyperintense signal seen in MR images of stroke immediately after ischemic onset in the brain has been a matter of debate since it was first demonstrated in 1990. In this article, we hypothesize and provide evidence that changes in the glial cells, comprising roughly one-half of the brain's cells and therefore a significant share of its volume, accompanying ischemia, are the root cause of the MRI signal change. Indeed, a primary function of the glial cells is osmoregulation in order to maintain homeostasis in the neurons and nerve fibers for accurate and consistent function. This realization also impacts our understanding of signal changes in other tissues following ischemia. We anticipate that this paradigm shift will facilitate new and improved models of MRI signals in tissues, which will, in turn, impact clinical utility.

## Introduction

Magnetic resonance imaging (MRI) is now a leading human imaging modality, which has revolutionized our study and understanding of the brain, in sickness and in health. Of its many capabilities in the diagnosis of human disease, MRI has proven crucial for the early detection of stroke and for guiding effective treatments (1). In particular, diffusion-weighted imaging (DWI) detects the tissue affected by stroke almost immediately after the infarct. This ischemic region, first demonstrated by Moseley et al. (2), appears as a hyperintensity on the DW image that represents a decrease in the overall apparent diffusion coefficient (ADC) of water in the affected tissue. This ischemic region develops over time as the stroke progresses and, at later times, also results in a change in the transverse relaxation time, *T*_2_ (3). In this article, we will be concerned with the initial diffusion changes and propose that glial cells drive the observed signal increase in acute stroke.

Currently, the origin of the observed diffusion signal increase in the infarct is still a matter of debate. Candidate mechanisms include changes in water exchange rates, membrane permeability, restricted diffusion effects, intra/extracellular compartmentation changes, and/or tortuosity effects potentially coupled with relaxation time differences between compartments—for a full discussion see Vestergaard-Poulson et al. (4), Hansen et al. (5). Neurite beading has also been suggested as a mechanism to explain the signal changes in the brain (6).

In order to uncover the likely mechanism(s) of these signal changes, MR microscopy was employed as a means for determining the origins of MR signals at the cellular level, albeit on very large cells initially (7). Since its inception three decades ago (7, 8), MR microscopy has continually improved, next being able to image the large L7 neuron from the sea slug *Aplysia californica* (9) and more recently applied to the study of isolated mammalian tissues at the single-cell level (10, 11).

This powerful methodology can be used to evaluate a hypothesis of the origin of the signal increase in DW images observed in stroke recently proposed by Le Bihan (12). He asserted that there is a restricted water pool near the cell membrane and that this slowly diffusing water pool would increase its volume as cells swell after an ischemic event. Curiously, the model involves only diffusion restriction on the inside of the cell membrane and not the outside. Moreover, if similar effects were to be applied externally, this would approximately double the volume of restricted water and change his conclusions significantly. Aside from that, MR microscopy studies of frog ova (6), *Aplysia californica* neurons (7), fixed animal (10), and human (11) neurons, and most recently live rat neurons (13) using MR microscopy methods, do not support this mechanism based upon membrane-bound water. If water diffusion was restricted at the cell membrane to the extent needed to affect the observed clinical signal changes seen in stroke, then DW images of these cells should include a relatively thick bright rim around the cell membranes where the restriction allegedly occurs. A bright rim of a size necessary to cause significant changes in volume fractions is not observed in any of the aforementioned cellular studies. Studies of erythrocyte ghosts also do not support this membrane-bound water hypothesis (14).

Alternatively, an intra/extracellular exchange mechanism was implied by Hsu et al. (15), following perfusion and tonicity studies performed in isolated *Aplysia californica* L7 neurons. In that work, following a 20% hypotonic perturbation, water diffusion in the cell cytoplasm remained constant, while *T*_2_ increased, indicating the cell was behaving as a perfect osmometer. This observation suggested that, after an ischemic event, the cells swell by absorbing water from the extracellular space. Since the ADC stays constant, and the ADC inside the cell was presumed slower than outside the cell, the resulting volume average of signals would result in an overall reduction of the ADC as observed on DW images of infracted areas in stroke.

However, recent microimaging of single neurons in fixed and live tissue contradicts this idea since the neurons are hypointense in DW images, and ADC maps clearly show that ADC in the cell body is larger than that outside the cell body (10, 11). This being the case, one would then expect the reverse to happen—i.e., the DW image of stroke tissue would show a *hypointense* signal at the lesion as neurons swell.

**Figure 1:**
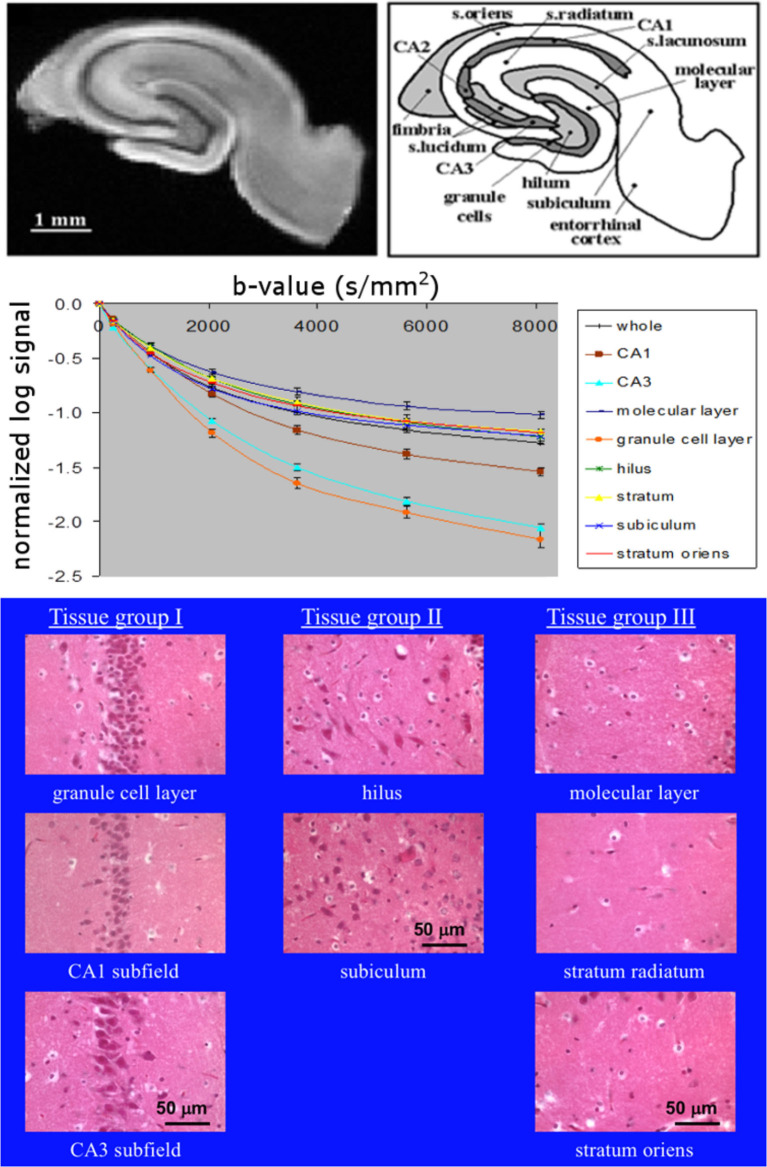
**Top**: MR microimage of a hippocampal brain slice (left) with a schematic of the anatomy (right) (reproduced with permission from (17) (Creative Commons license https://creativecommons.org/licenses/by/4.0/, the two elements unaltered but placed side by side), with experimental details in that publication). **Middle**: diffusion curves from anatomical regions (16). **Bottom**: grouped histological regions by neuronal density going from left to right (16).

This issue is further highlighted by data from our laboratory on excised, superfused brain slices. Diffusion signals obtained in segmented regions of the hippocampus correlated strongly with overall density of perikarya (16, 17) as observed in corresponding histology. Data from Shepherd et al. (17) is reproduced in modified form in Figure 1 and clearly shows regions rich in neuronal cell bodies to display faster signal decay than regions less densely populated by perikarya. Therefore, if the neuronal volume fraction was increasing, one would expect the ADC to increase, not decrease. In addition, one would expect perikarya-rich tissue like the stratum pyramidale in the hippocampus's *Cornu Ammonis* (CA1, 2, 3) regions would be hyperintense in the DW images when, in fact, they are hypointense (18), indicating again that the cell bodies have a faster ADC than the surrounding neuropil.

We are thus left with a conundrum. What is the origin of the hyperintensity in DW images in ischemic areas? Here, we present evidence for an alternative mechanism for the observed signal changes in stroke. In this perspective piece, we hypothesize and evidence that glial cells may be an important contributor to the observed signal changes.

## Discussion

The number of glial cells in the brain is still a subject of debate, with estimates ranging from 10 times that of the neurons to a number equivalent to that of neurons (19, 20). Given the brain's complexity and the large variation in the ratio of glia to neurons found in different subregions, calculation of these ratios and the volume contributions across the entire brain is a difficult and controversial issue (21). However, whatever the true volume, it is certain that the glial cells comprise a significant fraction of the total brain volume. A primary role of the glial cells is to maintain homeostasis—neurons and nerves thus maintain their water content for accurate and efficient operation.

**Figure 2:**
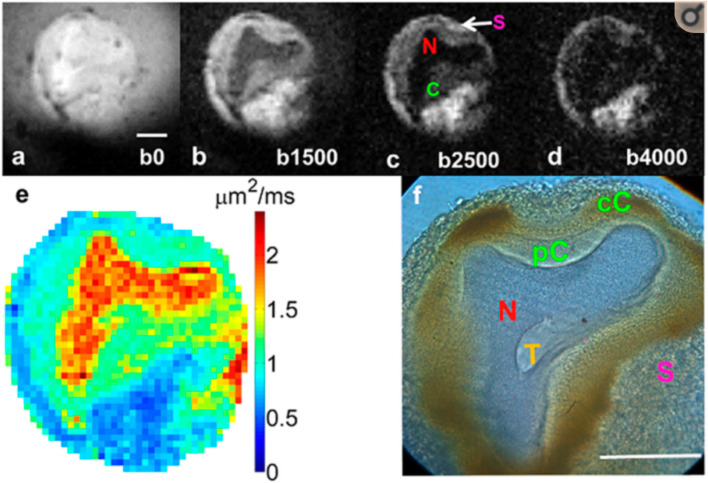
Diffusion-weighted images of an *Aplysia californica* L7 neuron at 4 b-values **(a–d)**. Notice that the labeled satellite cells (S) are hyperintense compared to the neuron. **(e)** Calculated diffusion map and **(f)** corresponding histology. Data reproduced with permission from Lee et al. (22)—experimental details are in that publication.

We hypothesize that the DW image changes observed at stroke onset are due to changes in the glial volume as these cells absorb water from the extracellular space and swell to maintain homeostasis of neurons. The resulting decrease in the ADC could then be explained provided that the diffusivity in the glial cells is lower than that of the neurons. A study by Lee et al. (22) on *Aplysia californica* neurons provides supporting evidence for this conjecture. In previous studies, the L7 neuron was stripped of surrounding tissue and cells by collagenase treatment; however, in Lee et al. (22), no collagenase was used, and the cell was extracted with the region containing satellite cells, mainly glia (23), intact. Figure 2 from that publication (22) shows that in a DW image, the signal from the region containing satellite cells is hyperintense compared to the neuron, itself, indicating that the ADC in the glia is lower than that in the neuron. These glial cells provide the necessary homeostasis for the neurons. Thus, after an infarct, the glia swell, and assuming the ADC within them also does not change with an osmotic perturbation, as implied by Hsu et al. (15), then the average ADC from the tissue will decrease as observed *in vivo*. This is because the glial volume increases with respect to the neuronal and extracellular volume. Glia swelling in stroke is supported by literature, and neuronal shrinkage has been observed to occur alongside glial swelling. In the study by Liu et al. (24), astrocytes were seen to have swelling after 90 min of ischemia, whereas in the same regions, the majority of neurons were shrunken by up to 25%. At 24 h post stroke, Garcia et al. (25) also observed swelling of astrocytes and shrinkage of neurons accompanied by initial axonal swelling and large hemispheric swelling (26). We, therefore, think it reasonable to assume that both neurons and extracellular water compartment contribute water to glial cells during ischemic events. We note here that these studies were not performed at the acute stage we are considering in this work, where we contend that the glia cells will be able to maintain the neuronal cell volume.

Since glial cells are ubiquitous in the nervous system, it would follow that the same mechanism will be evident in both gray and white matter. The axons of the white matter are myelinated making them much less permeable to water than the cell membrane. However the myelinated segments are interspersed with nodes of Ranvier that are approximately 1-μm length of unmyelinated axon membrane segments where ion and water exchange may occur. The length of the myelinated axolemma sections is dependent on the axon diameter (27). Further, there is evidence for water channels in the myelin sheath, itself (28). Whatever the mechanisms are, osmoregulation in axons is critical to ensure accurate and stable action potentials, whose conduction velocity is greatly accelerated by saltatory conduction (29). Again, osmoregulation in axon-rich brain tissue is affected by the glial cells. Thus, DW images should exhibit hyperintense signals during infarcts in both gray and white matter. At some point, the glial cells' capability for osmoregulation will be overwhelmed. This is supported by late-stage observations in rat brains exposed to sub-arachnoid hemorrhage (SAH): here, astrocyte volume was higher in SAH than in sham animals (30). We believe that at this point, vasogenic edema occurs, leading to measurable changes in *T*_1_ and *T*_2_ in the infarct. This potential causality between these events is the subject of future investigation.

We stress, here, that our discussion, thus far, has focused on the early stages of ischemia when the cells swell, and there is seen a hyperintensity in DW images, but no changes in *T*_2_. It is in this early stage that we will first develop and test the glial hypothesis in future studies. The signal changes following the acute phase will be time dependent and also dependent on other factors such as age, location of the stroke, size of the stroke, etc. (31). Later studies will also test the effectiveness of our biological models for assessing treatment, such as reperfusion, etc. (31), that are used in the clinic.

Although our data provide evidence for our hypothesis regarding glial cell osmoregulation and its effects upon diffusion MRI, direct confirmation is required in mammalian tissues. It is not yet possible to image glial cells with MR microscopy as they are too small. However, future studies may involve diffusion measurements on cultured mammalian glial cells assuming that the compartmental averaging issues can be addressed. Future studies and modeling of these systems at the single-cell level will also have to take account of the age of the specimens as demonstrated in maturation studies of *Aplysia californica* L7 neurons (32).

In addition to this glial hypothesis, Le Bihan (33) also published data showing that functional changes in brain tissue could be detected using DWI data acquired in humans. To our knowledge, other groups have not been able to reproduce this result, leading to speculation that Le Bihan et al. may be actually observing an artifact from the BOLD-fMRI contrast mechanism (34). However, it is important to note that studies in isolated buffer-superfused brain slices (35) devoid of blood do support possible changes in DW image signal associated with neuronal activity, as do studies on optic nerves (36) and spinal cord white matter (37). Specifically, if the glial hypothesis was correct, then we suggest that it is also changes in glial volumes that lead to the reduced ADC with neuronal activation in both buffer-perfused and blood-perfused preparation. We note here that Stanisz (38) included a glial compartment in his modeling approach to the issue of diffusion changes in the brain, recognizing that it would impact his results. However his estimate of the glial volume was too low at 17%, and he had no direct evidence on the diffusion in the glia relative to the neurons, nor perturbation studies at the cellular level, so could not come to the conclusion that we have in this work.

How then do we explain DW image changes associated with ischemia in non-neural tissues, which do not contain glial cells? MR microscopy studies of isolated perfused hearts with MR microscopy exposed to an ischemic event show an immediate decrease in the ADC (39) and *T*_2_ (40) after the ischemic insult. Without the osmoregulatory glial cells, the myocytes, themselves, will swell, leading to a change in the ADC and *T*_2_ with cell swelling in a straightforward intra/extracellular tissue model of water exchange. We suspect this will be the case in other non-neuronal tissues, and this will be a subject for future investigations. Cardiac studies were not performed at microscopic resolution because of the relatively large sample sizes (whole, excised rabbit hearts). However, microscopic resolution will be feasible on isolated heart slices (41) in the same manner in which brain slices have been imaged (Figure 1 in this paper). In this study on heart slices (41), the resolution was relaxed for SNR reasons to study multicomponent diffusion; however, since the sample size and equipment used is the same as that used for our Figure 1, MR microscopy will be feasible on the heart slices as well.

We note here that our discussion does hinge on using data from invertebrates to interpret rodent and clinical data. *Aplysia* is, indeed, an exceptionally well-used model for cells since theirs are large and accessible, so much so that the NIH funds an *Aplysia* facility in Miami to breed them for scientists. So far, studies of frog ova, *Aplysia* neurons, and mammalian tissues have produced broadly agreeing results with respect to intracellular water, i.e., reduced diffusion in the intracellular space compared to free water. In this work, it is assumed that the even slower diffusion in regions enriched with many glial cells, as observed in the satellite cells on the L7 neuron from *Aplysia californica*, will translate to mammalian tissues. Indeed, the very testing of the hypothesis presented here will be addressing this issue in future studies.

Caution is needed when trying to integrate results from cellular-level MR microscopy studies and preclinical MRI in animal models with the aim to interpret clinical data. Not only are the model systems employed in MR microscopy (frog eggs, isolated L7 neurons, acute brain slices, spinal cord, etc.) not directly comparable to the human brain but also the experimental details underlying the data are not comparable to clinical imaging. Extremely high SNR is required to achieve the image resolution needed to resolve individual cells. To achieve this, a combination of strong magnetic fields and minute (micro-)coils for radio frequency transmission and reception is typically used for MR microscopy on very small samples (7). Such setups are quite removed from clinical radiology. However, a more determining difference is related to the scanner gradient systems wherein MR microscopy and preclinical MRI gradients are typically strong (allowing short pulses), whereas for clinical DWI, diffusion gradient durations are typically an order of magnitude larger (on the order of TE/2). This is a problem because a lot of our current modeling assumes the short pulse approximation, which is rarely justified for clinical data. Furthermore, for longer diffusion times, water exchange likely cannot be ignored but largely is. The combined effects of diffusion time dependence, restriction sizes, exchange, etc., are difficult to untangle and likely quite different in MR microscopy and clinical MRI. Admittedly, this produces a situation where the comparison of MR microscopy to clinical MRI is complex. However, we point out that this is an issue common to all the microscopies (optical, confocal, X-ray, electron, atomic force, etc.), which interrogate small samples with specialized equipment not appropriate for direct clinical use. Care must be taken extending the results for clinical interpretation; for example, consider the sample preparation techniques required for electron microscopy (42). Still, we see MR microscopy as an essential tool to aid in understanding of clinical data, especially since the samples can be maintained as living tissue samples, allowing meaningful and informative perturbation studies to test models. We also point out that even if we can improve MRI to achieve true microscopic resolutions in the clinic, we will always be able to do much better on smaller samples with specialized equipment.

We end this perspective piece with a hefty dose of speculation or “what ifs.” Maintaining a stable tonicity in axons is essential for accurate and reproducible action potentials and, thus, fidelity of communication in the brain. What if for some reason the glial cells are compromised? What if they become hypo- or hypertonic? This may lead to tonicity changes in the axons, changing the velocity of the action potential. We appreciate that this is a complex issue—changes in tonicity also change axonal diameter, which also impacts conduction velocity (43). An increase in conduction velocity may be a factor in movement disorders where astrocytes have been linked with neuroinflammation and neurodegeneration (44), for example, in Parkinson's disease. A decrease in conduction velocity may be a factor in poor memory formation, again involving astrocytes (44), for example, in Alzheimer's. What if variations in glial tonicity lead to mood disorders, where glial cells have already been implicated to play a role (45, 46)? These ideas are very speculative, and we are not suggesting that glial cell dysfunction is a panacea for brain diseases. However, speculation along these lines may lead to fruitful avenues of research and potential clinical application in which MRI may play a major role. Replenishing the glial cells in some way may prove a potential treatment and seems feasible now that it has been demonstrated that they migrate when injected into the brain (47).

In conclusion, we have proposed and provide experimental support for the hypothesis that it is changes in glial cell volume that cause the ADC changes in ischemic brain tissue after stroke onset in both gray and white matter. Echoing a well-known statement on the relationship between mass and space, water tells glial cells how to change, and the glial cells tell water how to move, potentially solving a nearly 30-year-old mystery on the origin of the diffusion signal changes in stroke. With this in mind, new and accurate models of the tissue signals and how they evolve become possible. Other mechanisms will likely be involved in the resulting tissue changes to varying degrees; perturbation studies on model systems, both fixed and live, will facilitate the elucidation of these effects. It is our belief that this understanding will help improve the sensitivity and specificity of MRI for the diagnosis and monitoring of stroke, in particular, for distinguishing between reversible and irreversible ischemia and guiding the most effective course of personalized therapy. This understanding will also impact our interpretation of MR signals in both normal tissue and pathological tissues.

## Data Availability Statement

Publicly available datasets were analyzed in this study. This data can be found here: https://www.ncbi.nlm.nih.gov/pubmed/14663342, https://www.ncbi.nlm.nih.gov/pubmed/26059695.

## Author Contributions

JJF, TS, and CL performed the MR microscopy experiments described and processed image data and subsequent analysis. BH performed data processing and modeling. WS advised on glial cell biology. JRF advised on perfusion issues. SB originated the glial cell hypothesis and its consequences and was the PI and supervisor of studies from which results were reproduced, and wrote this manuscript. All authors contributed to the article and approved the submitted version.

## Conflict of Interest

The authors declare that the research was conducted in the absence of any commercial or financial relationships that could be construed as a potential conflict of interest.
